# Quantitative Comparison of Photoplethysmographic Waveform Characteristics: Effect of Measurement Site

**DOI:** 10.3389/fphys.2019.00198

**Published:** 2019-03-05

**Authors:** Vera Hartmann, Haipeng Liu, Fei Chen, Qian Qiu, Stephen Hughes, Dingchang Zheng

**Affiliations:** ^1^Faculty of Health, Education, Medicine and Social Care, Anglia Ruskin University, Chelmsford, United Kingdom; ^2^Department of Electrical and Electronic Engineering, Southern University of Science and Technology, Shenzhen, China

**Keywords:** multi-site PPG, photoplethysmography, PPG waveform analysis, pulse wave analysis, breathing pattern

## Abstract

**Introduction:** Photoplethysmography (PPG) has been widely used to assess cardiovascular function. However, few studies have comprehensively investigated the effect of measurement site on PPG waveform characteristics. This study aimed to provide a quantitative comparison on this.

**Methods:** Thirty six healthy subjects participated in this study. For each subject, PPG signals were sequentially recorded for 1 min from six different body sites (finger, wrist under (anatomically volar), wrist upper (dorsal), arm, earlobe, and forehead) under both normal and deep breathing patterns. For each body site under a certain breathing pattern, the mean amplitude was firstly derived from recorded PPG waveform which was then normalized to derive several waveform characteristics including the pulse peak time (Tp), dicrotic notch time (Tn), and the reflection index (RI). The effects of breathing pattern and measurement site on the waveform characteristics were finally investigated by the analysis of variance (ANOVA) with *post hoc* multiple comparisons.

**Results:** Under both breathing patterns, the PPG measurements from the finger achieved the highest percentage of analyzable waveforms for extracting waveform characteristics. There were significant effects of breathing pattern on Tn and RI (larger Tn and smaller RI with deep breathing on average, both *p* < 0.03). The effects of measurement site on mean amplitude, Tp, Tn, and RI were significant (all *p* < 0.001). The key results were that, under both breathing patterns, the mean amplitude from finger PPG was significantly larger and its Tp and RI were significantly smaller than those from the other five sites (all *p* < 0.001, except *p* = 0.04 for the Tp of “wrist under”), and Tn was only significantly larger than that from the earlobe (both *p* < 0.05).

**Conclusion:** This study has quantitatively confirmed the effect of PPG measurement site on PPG waveform characteristics (including mean amplitude, Tp, Tn, and RI), providing scientific evidence for a better understanding of the PPG waveform variations between different body sites.

## Introduction

Photoplethysmography (PPG) is a non-invasive and low-cost technique to measure blood volume change using an optical sensor. It provides useful physiological information to assess the cardiovascular function. PPG signals are commonly measured by the transmission and reflection methods which sense the light transmitted through or reflected by the tissue. Transmission method is applicable on body sites with thin tissue such as index finger while reflection method can be applicable on most of body sites (Joseph et al., 2014).

Although the origin of PPG waveform is still controversial and unconfirmed, various waveform characteristics have been extracted from the PPG signal and its derivatives, including the pulse peak time (Tp), dicrotic notch time (Tn), and reflection index (RI) (Wang et al., 2015), to reflect the general function of the systematic circulation and the interaction between left ventricle and peripheral vessels (Bahrain et al., 2014; Elgendi et al., 2018). These waveform characteristics have therefore been used for the estimation of arterial properties changes with different physiological conditions (Wang et al., 2015), the classification of systemic vascular resistance (Lee et al., 2011), and the diagnosis of various cardiovascular diseases (Elgendi, 2012).

The derivation of these waveform characteristics depends on the quality of the recorded PPG signals. It has been reported that PPG signals from different body sites have different signal quality (Hernando et al., 2017) and waveform shape (Nilsson et al., 2007). The aforementioned waveform characteristics (Tn, Tp, and RI) might therefore be influenced by the measurement site of PPG. The difference in PPG waveforms between the commonly used measurement body sites (finger, forehead, toe, and earlobe) has been investigated to guide clinical diagnosis and treatment (Sharkey et al., 2018; Sun et al., 2018). The multi-site PPG system, in which PPG signals are measured simultaneously from finger, toe, and earlobe, has been developed to investigate the peripheral arterial disease (Bentham et al., 2018), the Raynaud’s phenomenon and systemic sclerosis (McKay et al., 2014), and Takayasu’s arteritis (Lakshmanan et al., 2018). However, to the best of our knowledge, no studies have quantitatively and comprehensively investigated the effect of measurement site on the waveform characteristics derived from PPG signals recorded from the same optical sensor.

PPG waveform also changes with breathing pattern. The amplitude, frequency, and baseline of PPG waveform are modulated by respiration (Pimentel et al., 2015). Compared with normal breathing, slow, and deep breathing enhances the amplitude fluctuations of PPG signal (Yuan et al., 2018). The interaction between breathing pattern and measurement site might influence the PPG waveform, but has not been fully investigated.

This study aims to provide a quantitative investigation of the effects of measurement site and breathing pattern (normal and deep breathing) on the PPG waveform characteristics (mean pulse amplitude, Tp, Tn, and RI).

## Materials and Methods

### Subjects

Thirty six healthy subjects (24 female and 12 male, mean ± SD of age: 32.7 ± 12.3 years) were recruited in the study. They gave written informed consent to participate in the study. None of the volunteers had known cardiovascular diseases. The protocol was approved by the Research Ethics Committee of the Faculty of Medical Science, Anglia Ruskin University, United Kingdom. Table 1 gives an overview of basic subject information, including age, weight, and height.

**Table 1:** Basic subject information including age, weight, height, and resting blood pressures.

Subject information					
No. subjects	36				
No. male	12				
No. female	24				

		**Mean**	**Min**	**Max**	**SD**

Age (years)		33	19	58	12
Weight (kg)		70	45	90	12
Height (cm)		170	154	186	8
SBP (mmHg)		117	93	172	14
DBP (mmHg)		77	58	98	9


### Measurement Protocol and Procedure

The experiments were performed in a quiet measurement room at Anglia Ruskin University. After a 10-min relaxation in a seated position, resting systolic, and diastolic BP values (SBP and DBP) were measured using a clinically validated automatic BP monitor (HEM-7322U-E from Omron healthcare) (Table 1). Subsequently, the subjects were asked to lie down on a comfortable clinical measurement bed for PPG recording.

**FIGURE 1:**
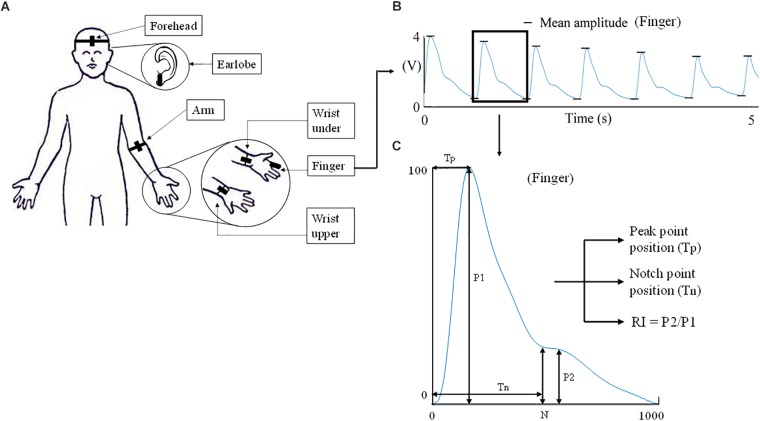
Experimental protocol of PPG waveform recording and definition of waveform characteristics. **(A)** The six measurement sites (finger, wrist under, wrist upper, arm, earlobe, and forehead). **(B)** A 5-s segment of the waveform measured from finger. The extraction of mean amplitude is also illustrated. **(C)** The characteristics derived from the normalized PPG waveform.

A reflective PPG sensor was used in this study. The sensor was developed with an identical pair of surface-mount emitting diode (SME 2470-001, Honeywell) and photodiode (SMD 2420-001, Honeywell). The SME2470 is a high intensity aluminum gallium arsenide infrared emitting diode, which has a beam angle of 24 degree. The output peak wavelength of the emitting diode is about 880 nm, which matches with the maximum photosensitivity wavelength of the SMD2420 photodiode, and supplies optimum optical characteristics and efficient optical coupling. The PPG sensor was placed sequentially on different body sites (finger, wrist under, wrist upper, arm, earlobe, and forehead, as shown in Figure 1). The sites on the volar and dorsal sides of the wrist were named as “wrist under” and “wrist upper” for brief. A finger clip, ear clip or Velcro fastener were used to fix the sensor on the finger, ear and other sites. The measurement order of the body sites was randomized. During the whole measurement, the participants were asked not to talk or move to reduce the potential effect of motion artifacts on the quality of PPG signals.

Considering that different breathing patterns may influence the effect of measurement site on PPG waveforms, PPG signals from different measurement sites were recorded under resting (normal breathing) and deep breathing patterns in this study. The first measurement session (six measurements) was conducted under normal breathing condition. The second session (additional six measurements) was under deep breathing condition. Normal breathing was defined as a subject’s own normal breathing behavior. Deep breathing was fulfilled by following a paced breathing app (Paced breathing, Trex LLC) with a defined period of each 5 s for both inhalation and exhalation.

**FIGURE 2:**
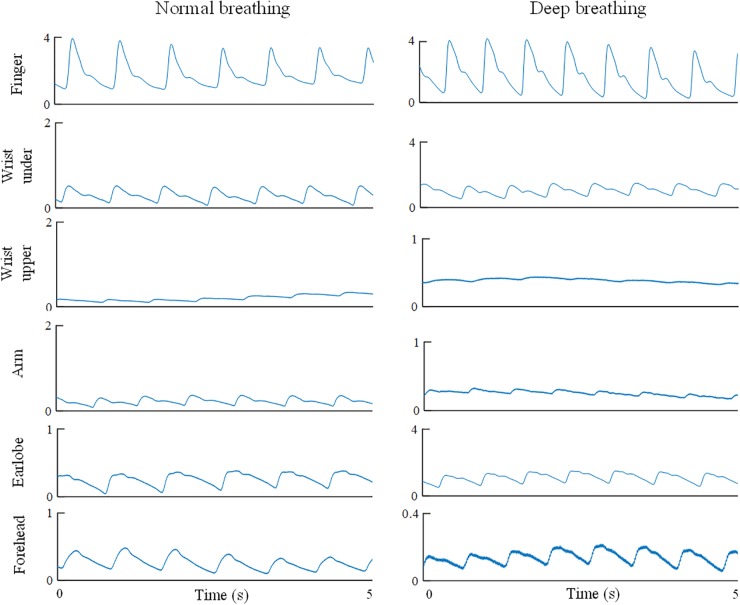
Illustration of recorded PPG waveforms of one subject from different body sites under both normal and deep breathing patterns. Five-second segments were extracted from the 1-min recording.

All the PPG waveforms were acquired and digitally recorded by the MP160 Data acquisition system using the Biopac AcqKnowledge software. Each PPG recording from one body site lasted for 1 min with a 1-min gap between recordings. In total, 12 recordings were obtained from each subject (from six sites under two breathing patterns). Figure 2 gives the examples of recorded raw PPG waveforms (5-s segments extracted from the 1-min recording) from the six body sites of a subject under both breathing patterns.

### PPG Waveform Analysis

**FIGURE 3:**
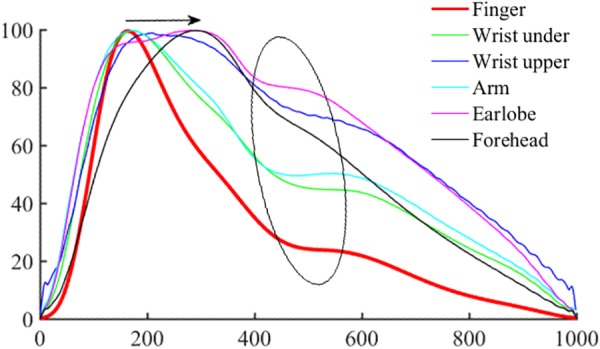
Normalized and averaged PPG waveforms of one subject from the six body sites under normal breathing pattern.

For each raw PPG waveform, the pulse amplitude was defined as the difference between the maximum (systolic) and minimum (end-of-diastolic) values within a cardiac cycle. The mean amplitude was calculated as the average of all the pulses of a single recording (Figure 1B). The raw PPG waveform from each measurement site was then normalized as follows: firstly, its baseline drift was removed. Secondly, all the pulses within a single recording were normalized beat-by-beat in both width (1000 sampling points) and amplitude (0–100) after the foot detection of each pulse (Figure 1C). Thirdly, all the normalized pulses were averaged to get a single reference normalized waveform for each body site, as shown in Figure 1C. Figure 3 shows the examples of normalized and averaged PPG waveforms from the six body sites of one subject under normal breathing pattern.

Waveform characteristics were then derived from each normalized PPG waveform. Tp and Tn were calculated from the end-of-diastole to the positions of systolic peak and dicrotic notch (Figure 1C). The locations of the systolic peak and dicrotic notch points were associated with the first and second zero-crossing points of the first derivative of PPG signal. Manual check was also performed to ensure the accurate identification. RI was calculated as the ratio between reflection peak amplitude (P2) and pulse maximum amplitude (P1) (Wang et al., 2015).

### Statistical Analysis

The mean and standard deviation (SD) of each waveform characteristics (mean amplitude, Tp, Tn, and RI) were calculated across all the subjects, separately for the six measurement sites and for the two breathing patterns. To quantitatively investigate the effect of measurement site, breathing pattern, gender and age on waveform characteristics, analysis of variance (ANOVA) with *post hoc* multiple comparisons were performed on SPSS (Version 24.0, IBM Corp.) to examine if there were significant differences in mean amplitude, Tp, Tn, and RI between male and female, between the various measurement sites (referred to the finger), and between two breathing patterns, as well as the effect of age. The criterion of statistical significance was *p* < 0.05 for all waveform characteristics.

## Results

### Analyzability of PPG Signals to Derive Waveform Characteristics

In total, 432 recordings were obtained (from 6 measurement sites, 2 breathing conditions, and 36 subjects). The mean amplitude could not be detected from 12 recordings (5 under normal breathing, 7 under deep breathing), less than 3% in total. Based on the normalized PPG waveforms, generally, PPG waveform characteristics were more analyzable under normal breathing than deep breathing. Tp could not be determined in 10 recordings (5 during normal breathing, 5 during deep breathing), which was about 2% of total recordings. Tn and RI values could not be derived from approximately 24% of total recordings (104 recordings, 36 under normal breathing, 68 under deep breathing, respectively).

Regarding the difference in overall analyzability (all the waveform characteristics considered) between different measurement sites, under normal breathing, finger produced the most analyzable PPG waveforms (95%, 34 out of 36 recordings with all the four characteristics analyzable), followed by wrist under (86%), arm (83%), earlobe (81%), wrist upper (67%), and finally forehead (61%). Under deep breathing the best site was still the finger (86% analyzable), followed by wrist under (78%), earlobe (75%), arm (70%), wrist upper (61%), and forehead (42%). Therefore, the finger and forehead were the best and worst measurement sites to derive analyzable waveform characteristics under both breathing patterns.

### Effect of Measurement Site on Mean Amplitude

**FIGURE 4:**
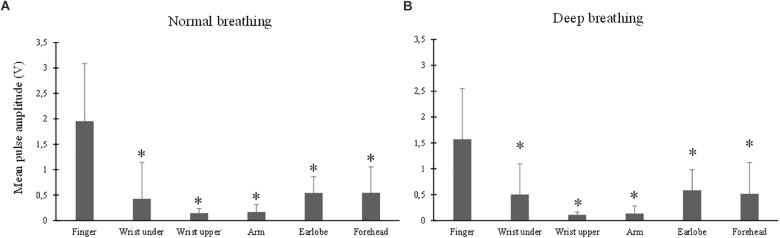
Mean amplitude of PPG waveform measured from different sites under both normal **(A)** and deep **(B)** breathing patterns. ^∗^Marks the significant differences in comparison with that from the finger (*p* < 0.05).

The ANOVA results showed that the effect of breathing pattern on mean amplitude was insignificant (*p* > 0.05) while the effect of measurement site was significant (*p* < 0.001). Figure 4 shows the mean amplitude level (in V) from different measurement sites under normal (A) and deep (B) breathing patterns. Under both breathing patterns, the mean amplitude from finger PPG was significantly higher than those from other sites (all *p* < 0.001). The lowest mean amplitude was obtained from the “wrist upper” PPG.

### Effect of Measurement Site on Tp

**FIGURE 5:**
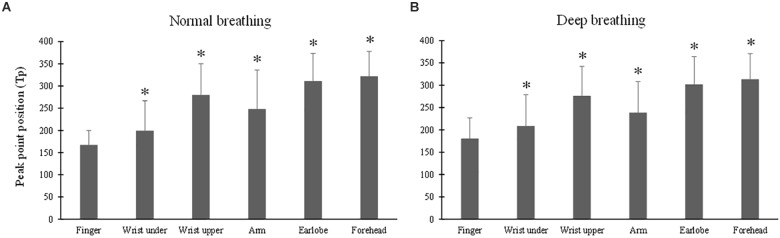
Photoplethysmography pulse peak point position (Tp) measured from different sites under normal **(A)** and deep **(B)** breathing patterns. ^∗^Marks the significant difference in comparison with that from the finger (*p* < 0.05).

The ANOVA results of Tp indicated that the effect of breathing pattern was insignificant (*p* > 0.05) while the effect of measurement site was significant (*p* < 0.001). Figure 5A,B show that, under both breathing conditions, Tp of the finger PPG was significantly smaller than those of all the other sites (all *p* < 0.001, except *p* = 0.04 for “wrist under”). The highest Tp values were derived from the forehead PPG waveforms.

### Effect of Measurement Site on Tn

**FIGURE 6:**
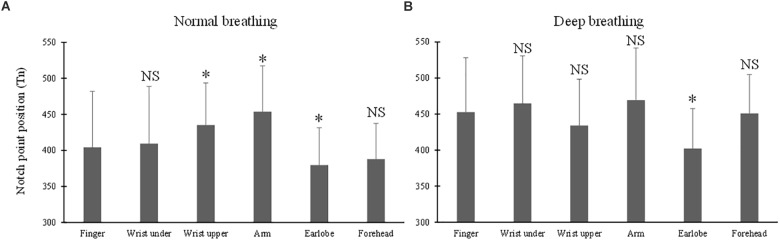
Dicrotic notch point position (Tn) measured from different sites under both normal **(A)** and deep **(B)** breathing pattern. ^∗^Marks the significant difference in comparison with that from the finger (*p* < 0.05). NS means no significant difference in comparison with that from the finger (*p* > 0.05).

The ANOVA results of Tn indicated the significant effects of breathing pattern and measurement site (both *p* < 0.001). Under normal breathing pattern (Figure 6A), Tn from the finger PPG was not significantly different from those of the wrist under and forehead (*p* = 0.7 and 0.2), but was significantly different from those of the other three sites (*p* < 0.001 for the arm, *p* = 0.02 for the wrist upper and *p* = 0.04 for the earlobe). Under deep breathing pattern (Figure 6B), only Tn from the earlobe was significantly smaller than that from the finger (*p* < 0.001).

### Effect of Measurement Site on RI

**FIGURE 7:**
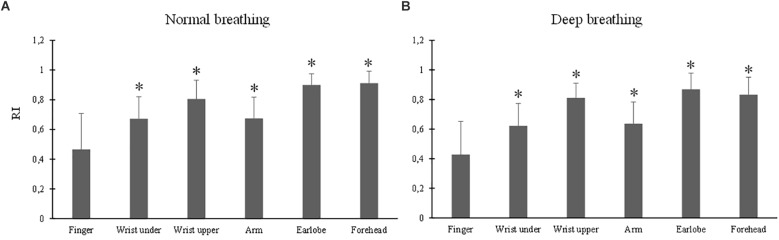
Reflection index values measured from different sites under normal **(A)** and deep **(B)** breathing patterns. ^∗^Marks the significant differences in comparison with that from the finger (*p* < 0.05).

The ANOVA results of RI indicated the significant effects of breathing pattern (*p* = 0.02) and measurement site (*p* < 0.001). Under both breathing patterns, the RI values from all the other sites were significantly larger than that from the finger (all *p* < 0.001) (Figure 7). The largest RI was obtained from the forehead PPG.

### Effects of Gender and Age on the Waveform Characteristics

The ANOVA results showed insignificant effect of gender on mean amplitude, Tp, Tn and RI (all *p* > 0.05). The effect of age was statistically insignificant on mean amplitude (*p* > 0.05), but significant on Tp (*p* < 0.01), Tn (*p* < 0.001), and RI (*p* < 0.001).

## Discussion and Conclusion

To the best of our knowledge, this is the first study focusing on the quantitative investigation of the effect of measurement site on the waveform characteristics (mean amplitude, Tp, Tn, and RI) of PPGs recorded from the same optical sensor.

A clear PPG signal is important for the analysis of its waveform characteristics (Allen and Murray, 2004). This study has concluded that, under both breathing conditions, the measurement sites of finger and earlobe produced more analyzable PPG signals. Finger and earlobe could therefore be recommended as relatively better measurement sites for deriving identifiable waveform characteristics. This was partially due to the rich arterial supply and the relative convenience to affix sensors (Sun and Thakor, 2016). Secondly, different body sites differ in their skin pigmentation and tissue thickness which influence the waveform shape of the recorded PPG signals (Nilsson et al., 2007). The cutaneous vascular walls of the finger are richly innervated by α-adrenoceptors, resulting in higher sensitivity to the volumetric fluctuations of blood than other body sites (Alian and Shelley, 2014) including the earlobe (Charlton et al., 2017). Therefore, in real practice, the finger is the most commonly applied body site for PPG measurement, considering its reliable measurement of arterial pulsation and its convenience of use (Alian and Shelley, 2014). It has also been observed in this study that forehead derived the least analyzable PPG signals under both normal and deep breathing patterns. Forehead PPG waveform is relatively smoother in the diastole phase, generating difficulties for identifying the notch point position (Peralta et al., 2017).

In comparison with normal breathing, under deep breathing condition the percentage of PPG signals that could not be used for deriving waveform characteristics was relatively higher. This might be related to some physiological factors. Firstly, the respiration influences the PPG waveform by baseline wandering, amplitude modulation, and frequency modulation (Pimentel et al., 2015). Under deep breathing, the enlarged differences in oxygen saturation between inhalation and exhalation enhance the differences of PPG waveforms between cardiac cycles, therefore increasing the difficulty in waveform normalization and parameter analysis. Secondly, the myogenic and neurogenic fluctuations of 0.05–0.15 Hz, and the noises of 0.1–0.2 Hz commonly influence the PPG signal during deep breathing, especially on the waveforms recorded from the forehead (Hernando et al., 2017).

It has been accepted that Tp, Tn, and RI can be used for the diagnosis of vascular diseases (Qawqzeh et al., 2010; Fangming et al., 2014). Tn reflects the transmission of reflective pulse wave. RI indicates the amplitude of reflective pulse wave as well as the changes in vasomotor tone, particularly the occurrence of vasodilation (Lee et al., 2011). The reflective pulse wave varies in amplitude, velocity, and arrival time between different measurement sites, forming different PPG waveforms when composed to the forward PPG wave. It has been reported that the maximal oxygen uptake had a significant effect on the arterial properties (quantified by Tp, Tn, and RI derived from the finger PPG waveforms) of athletes (Wang et al., 2015). Accordingly, in our results, the effect of breathing pattern on Tp was negligible, but observable on Tn and RI, reflecting the physiological cardiorespiratory influences on the PPG waveform.

Importantly, this study has demonstrated that measurement site had significant effects on the pulse waveform characteristics. The mean amplitude of PPG signal from the finger, and those from the earlobe and forehead, composed the highest and lowest values. In parallel studies, the finger derived higher PPG waveform amplitude than the wrist and arm. It was deduced that peripheral areas have large vascular bed and consequently higher PPG amplitude (Maeda et al., 2011). The smaller Tp at the finger compared with other sites was in accordance with existing studies in which shorter pulse rise time was observed in PPG waveforms from peripheral sites such as the finger and toe (Allen and Murray, 2000; Sharkey et al., 2018). The RI from finger PPG was significantly smaller than those of other sites. The P2 of a PPG waveform reflects the superimposed reflection pulse waves from multiple arterial bifurcations (Rubins, 2008). As a peripheral arterial end, the finger has few reflection pulse waves and consequently low RI. Due to the proximity to the heart with high vascularity and therefore a lower total resistance to flow over the capillary bed, the PPG waveforms from head and earlobe have smoother systolic peaks (Sharkey et al., 2018), accounting for the higher RIs (Figure 7). Considering the observed significant effect of measurement site on all the pulse waveform characteristics, the measurement site is therefore an important factor when analyzing waveform characteristics for different clinical applications.

In this study, not every site available for PPG signal measurement was included. PPG signal from the sternum site has been attempted in the published studies (Chreiteh et al., 2015; Finkelstein et al., 2017) but not in the formal experiment of this study. The PPG sensors applied on the sternum site were mainly based on green light (Finkelstein et al., 2017). Chreiteh et al’s. (2015) study developed advanced circuit to collect PPG signal from sternum site with infrared sensor. Furthermore, these published studies mainly focused on the estimation of pulse rate or its variability, and breathing rate, not on the analysis of waveform characteristics. However, with the main focus of our study to compare the waveform characteristics from the same PPG optical sensor, the recording of clear PPG waveform is required. Therefore, only the six sites with good signal quality were finally selected in this study.

The main limitation of this pilot study is that only 36 healthy subjects were included. In the future, a large-scale population study would be useful to confirm the results in different physiological conditions. Although it is not the main focus of the current study to investigate the effect of aging on PPG waveform, as an important factor that influences PPG waveform and derived waveform parameters (Allen and Murray, 2003), the interactive relationship between aging and measurement site on PPG waveform characteristics could be investigated in future studies. Limited by the conditions, it was difficult to keep non-stationary positions during the whole process of measurement in six body sites. More dynamic conditions could be considered in future studies based on more advanced PPG sensors. The investigation should also be conducted on subjects with cardiovascular and other related diseases to investigate the different effect of body sites on PPG waveform under pathological conditions. Nevertheless, as a pilot study, considering the obviously different PPG waveforms from various body sites (Nilsson et al., 2007), the current study paves the way for the detailed understanding of local PPG waveform characteristics.

In conclusion, this study has quantitatively concluded that the measurement site had a significant effect on PPG waveform parameters, providing quantitative information to better understand the underlying mechanism of waveform shape from different body sites.

## Data Availability

All datasets generated for this study are included in the manuscript and/or the supplementary files.

## Ethics Statement

This study was carried out in accordance with the recommendations of the Local Research Ethics Committee of the Faculty Research Ethics Panel (FREP) under the terms of Anglia Ruskin University with written informed consent from all subjects. All subjects gave written informed consent in accordance with the Declaration of Helsinki. The protocol was approved by the Local Research Ethics Committee of the Faculty Research Ethics Panel (FREP) under the terms of Anglia Ruskin University.

## Author Contributions

VH performed most of the original experiments described in this study. VH, HL, and QQ analyzed the data. All authors contributed to drafting of the manuscript and the discussion and concur with the submitted version of the manuscript. DZ and FC supervised the project that led to production of the results shown, and critically reviewed and edited the manuscript.

## Conflict of Interest Statement

The authors declare that the research was conducted in the absence of any commercial or financial relationships that could be construed as a potential conflict of interest.

## Abbreviations

AC: alternating current
ANS: autonomic nervous system
DBP: diastolic blood pressure
DC: direct current
FREP: Faculty Research Ethics Panel
NS: not significant
P1: pulse maximum amplitude
P2: reflection peak amplitude
PPG: photoplethysmography
RI: reflection index
SBP: systolic blood pressure
Tn: dicrotic notch time
Tp: pulse peak time
